# Incidence and serotype distribution of invasive group B streptococcal disease in young infants: a multi-country observational study

**DOI:** 10.1186/s12887-015-0460-2

**Published:** 2015-10-01

**Authors:** Luis Rivera, Xavier Sáez-Llorens, Jesus Feris-Iglesias, Margaret Ip, Samir Saha, Peter V. Adrian, Shabir A. Madhi, Irving C. Boudville, Marianne C. Cunnington, Javier M. Casellas, Karen S. Slobod

**Affiliations:** Hospital Maternidad Nuestra Señora de la Altagracia, Santo Domingo, Dominican Republic; Hospital del Niño and the School of Medicine of the University of Panama, Panama City, Panama; Hospital Infantil Dr. Robert Reid Cabral, Santo Domingo, República Dominicana; Department of Microbiology, Chinese University of Hong Kong, Hong Kong, SAR China; Department of Microbiology, Bangladesh Institute of Child Health, Dhaka Shishu Hospital, Dhaka, Bangladesh; Medical Research Council Respiratory and Meningeal Pathogens Research Unit, University of the Witwatersrand, Johannesburg, South Africa; National Institute for Communicable Diseases, Sandringham, South Africa; Novartis Vaccines and Diagnostics Inc., Cambridge, MA USA; Global Development, Novartis Vaccines and Diagnostics, Frimley Business Park, Frimley, Camberley, Surrey GU16 7SR UK

**Keywords:** Group B streptococcal disease, Incidence, Serotype distribution, Asia, Latin America

## Abstract

**Background:**

Group B Streptococcus (GBS) is a leading cause of serious infection in very young infants. Robust incidence data from many geographic regions, including Latin America and Asia, are however lacking.

**Methods:**

A multicenter, hospital-based observational study was performed in Panama, Dominican Republic, Hong Kong and Bangladesh. All represented urban, tertiary referral hospitals, except Bangladesh. GBS cases (microbiological isolation from normally sterile sites in infants aged 0–89 days) were collected over 12 months.

**Results:**

At 2.35 (95 % CI: 1.74–3.18) cases per 1000 live births, the incidence of early onset GBS disease (EOD) was highest in the Dominican Republic, compared with 0.76 (95 % CI: 0.41–1.39) in Hong Kong and 0.77 (95 % CI: 0.44–1.35) in Panama, while no cases were identified in Bangladesh. Over 90 % of EOD cases occurred on the first day of life, with case fatality ratios ranging from 6.7 % to 40 %, varying by center, age of onset and clinical presentation. Overall, 90 % of GBS (EOD and late onset disease) was due to serotypes Ia, Ib and III.

**Conclusions:**

The incidence rate of early onset GBS infection reported in Dominican Republic was not dissimilar from that described in the United States prior to screening and intrapartum antibiotic prophylaxis, while the incidence in Hong Kong was higher than previously reported in the Asian region. The failure to identify GBS cases in Bangladesh highlights a need to better understand the contribution of population, healthcare and surveillance practice to variation in reported incidence. Overall, the identified disease burden and serotype distribution support the need for effective prevention methods in these populations, and the need for community based surveillance studies in rural areas where access to healthcare may be challenging.

## Background

Group B Streptococcus (GBS) or *Streptococcus agalactiae* is a significant cause of serious infections in neonates, manifesting as sepsis, pneumonia and meningitis. GBS is commonly identified in rectal and vaginal cultures of women who are colonized, a recognized risk factor for perinatal infection [1]. Infant GBS infection occurs as a continuum over the first 3 months of life, but has often been categorized as early-onset disease (EOD; occurring between birth and 6 days of age) or late-onset (LOD; occurring between 7 and 89 days of age) [1]. Five of nine capsular serotypes, III, Ia, V, Ib and II, cause 95 % of invasive disease (in decreasing frequency) [2].

A recent meta-analysis of published studies reported a mean global incidence of 0.53 per 1000 live births (LB), with a range from 0.02 per 1000 LB in Southeast Asia to 1.21 per 1000 LB in Africa (data primarily from Southern and Eastern Africa). Incidence data from Asia were based on relatively few studies [2]. Such variation may reflect differences in study methodology (specifically, in case ascertainment and laboratory diagnostics), antibiotic usage, health care access or possibly regional population differences [3]. The wide variation in the reported perinatal GBS incidence contrasts with the more consistent reported rates of maternal GBS colonization [4]. This incongruity mandates a strong focus on case detection methodology in prospective surveillance studies.

Where implemented, administration of intrapartum antibiotic prophylaxis (IAP) to mothers at risk of delivering GBS-infected infants (defined by maternal colonization or specified clinical risk factors) has reduced but not eliminated EOD, and has had no effect on LOD [5–7]. Implementation of IAP remains difficult in resource-constrained regions due to logistics and cost, and requires routine access to healthcare. A maternal vaccine against serotypes Ia, Ib and III is also in development [8]. Robust global data demonstrating the incidence and serotype distribution of GBS infection in young infants will be invaluable to understand the potential impact of these preventive measures [9]. The current observational study evaluated the incidence, serotype distribution and case fatality ratio (CFR) of invasive GBS disease in infants <3 months in three hospitals in Latin America and two hospitals in Asia, where few data currently exist.

## Methods

### Study design and setting

This was an observational, multi-center study performed in five hospitals in four countries (Panama, Dominican Republic [two hospitals], Hong Kong, Bangladesh). All study hospitals were large urban referral centers except in Bangladesh where the facility was a rural, not-for-profit private hospital). Study hospitals offered maternity services, for both low and high risk pregnancies with skilled attendants, as well as pediatric services. The exception was in Dominican Republic where two hospital centers participated: the first provided maternity and neonatal services only and the second provided pediatric services. The hospitals were within close proximity of each other serving the same catchment population.

The study hospital in Panama was a large maternity hospital in the capital city with 15000 births per year. Infants developing signs and symptoms of sepsis were immediately referred to the national pediatric reference hospital which was directly adjacent to the maternity hospital. The first hospital center in Dominican Republic served as the national reference for maternal medicine with 18000 deliveries per year. Although, neonatal intensive care services were available on site; the hospital had no pediatric services. A second hospital, offering pediatric and neonatal services for the same catchment population in the capital, therefore participated to ensure the capture of GBS LOD cases presenting post maternal discharge. The site in Hong Kong included public hospitals that offered obstetric, neonatal and pediatric services for a densely populated urban area with a catchment of 1.3 million inhabitants with 13000 deliveries per year. Over 90 % of births occur in hospital in Panama, Dominican Republic and Hong Kong.

The study hospital in Bangladesh was one of two hospitals serving the rural sub-district of Mizapur in Bangladesh. It is a private, not for profit hospital with both obstetric and pediatric services and 8000 deliveries per year. Previous studies reported that only 25 % of births in the Mirzapur region occur in hospital [10].

All study centers implemented risk-based screening for GBS as standard of care: mothers presenting in labor with clinical risk factors associated with increased risk of GBS disease in the infant (e.g., maternal fever, prolonged rupture of the membranes) were administered intravenous antibiotic prophylaxis.

The study protocol was approved by local ethics committees and conducted in compliance with Good Pharmacoepidemiological Practice, local regulations and the Declaration of Helsinki (2008). This study was approved by the following ethics committees: the Comite de Bioetica en la Investigacion del Hospital del Niño and the Comite Nacional de Bioetica en la Investigacion del Instituto conmemorativo Gorgas de Estudios de la Salud in Panama, the Comite de Bioetica del Hospital Maternidad Nuestra Señora de la Altagracia and the Comite de Etica y de Investigaciones Fundacion Dominicana de Infectologia, Inc in Dominican Republic, the Joint Chinese University of Hong Kong – New Territories East Cluster Clinical Research Ethics Committee and the Ethical Review Committee of the Bangladesh Institute of Child Health.

Written informed consent was obtained from the parents/guardians of all subjects. The informed consent was countersigned by the personnel who had conducted the informed consent discussion who was qualified according to local regulations.

### Study population

Infants aged 0 to 89 days, were enrolled into the study following microbiological confirmation of GBS by a positive culture from a sterile site (blood, cerebrospinal fluid, lung aspirate, joint fluid) according to local laboratory procedures, and informed consent from the legal parent/guardian (Enrolled Population). Sterile site cultures were routinely performed, according to local standards, on all infants admitted with clinical signs and symptoms of sepsis, meningitis or pneumonia. Enrollment was primarily prospective at the time of diagnosis. Given the rare primary outcome, retrospective enrollment was permitted by the protocol. Infant follow up was from the time of enrolment until the first of hospital discharge, death or withdrawal from the study. The study duration (enrollment period) was 12 months at each study hospital.

### Data collection and laboratory methods

Data concerning subject demographics, birth and disease characteristics, disease outcome, maternal and infant antibiotic use were extracted from medical records and/or parental/guardian interviews following subject enrollment.

The microbiological confirmation of GBS followed local standards, though all centers used automated (BacT/Alert®; Biomerieux, France or BACTEC®; Becton Dickinson, United States) enrichment culture methods. Suspected Group B Streptococcus colonies were confirmed using Gram stain and GBS antigen latex agglutination testing or biochemical (Christie Atkinson Munch-Petersen, aesculin bile) testing. Serotyping of recovered GBS isolates was performed using the Strep-B-Latex™ (Statens Serum Institut, Denmark) rapid latex agglutination test [11] in one central laboratory (The Respiratory and Meningeal Pathogens Research Unit, Johannesburg, South Africa).

### Sample size

As an observational, descriptive study with no *a priori* hypothesis, there was no pre-defined sample size. However, the precision of incidence estimates depended on the underlying GBS incidence and birth cohort size. If five GBS cases were observed among 10,000 births, the point incidence estimate would be 0.5 per 1000 LB with a 95 % confidence interval (CI) of 0.16 to 1.17 per 1000 LB. For the same incidence, a birth cohort of 20,000 LB would give increased precision with a narrower 95 % CI of 0.24 to 0.92 per 1000 LB.A birth cohort of 5,000 would give reduced precision with a 95 % CI of 0.05 to 1.44 per 1000 LB. The annual birth cohort within the study varied from 4,227 in Bangladesh, to 17,867 in Dominican Republic.

### Statistical methods

To be eligible for analysis (Analysis Population), enrolled infants were required to be born in the study hospital (all centers with maternity unit) or within the catchment area for the study hospital (centers without a maternity unit). Demographics and baseline characteristics of the Analysis Population were summarized descriptively using the mean (standard deviation) or median (range) for continuous variables and the frequency distribution for categorical variables. The incidence rate was expressed as rate per 1000 live births (LB), and computed for each hospital center $\left\{i=\frac{total\ n{}^{\circ}\  of\  cases\  admitted\  from\  study\  start\ to\ 12\  months}{total\ n{}^{\circ}\  of\  live\  births\  from\  study\  start\ to\ 12\  months}\times 1\ 000\right\}$.

Denominators for incidence calculation used: (i) the in-hospital birth cohort for centers with a maternity unit, or (ii) the number of LB occurring in the community catchment area of the hospital for non-maternity centers. Serotype distribution was described for the Analysis Population as a frequency distribution (number and percentage of participants) for EOD, LOD and total cases. The case fatality ratio (CFR) was expressed as the percentage (%) of GBS cases in the Analysis Population who died due to GBS invasive disease within the study period. The Wilson score interval method was used to calculate all 95 % CI [12]. All analyses were completed using SPSS version 20.

## Results

### Characteristics of study population

**Fig. 1:**
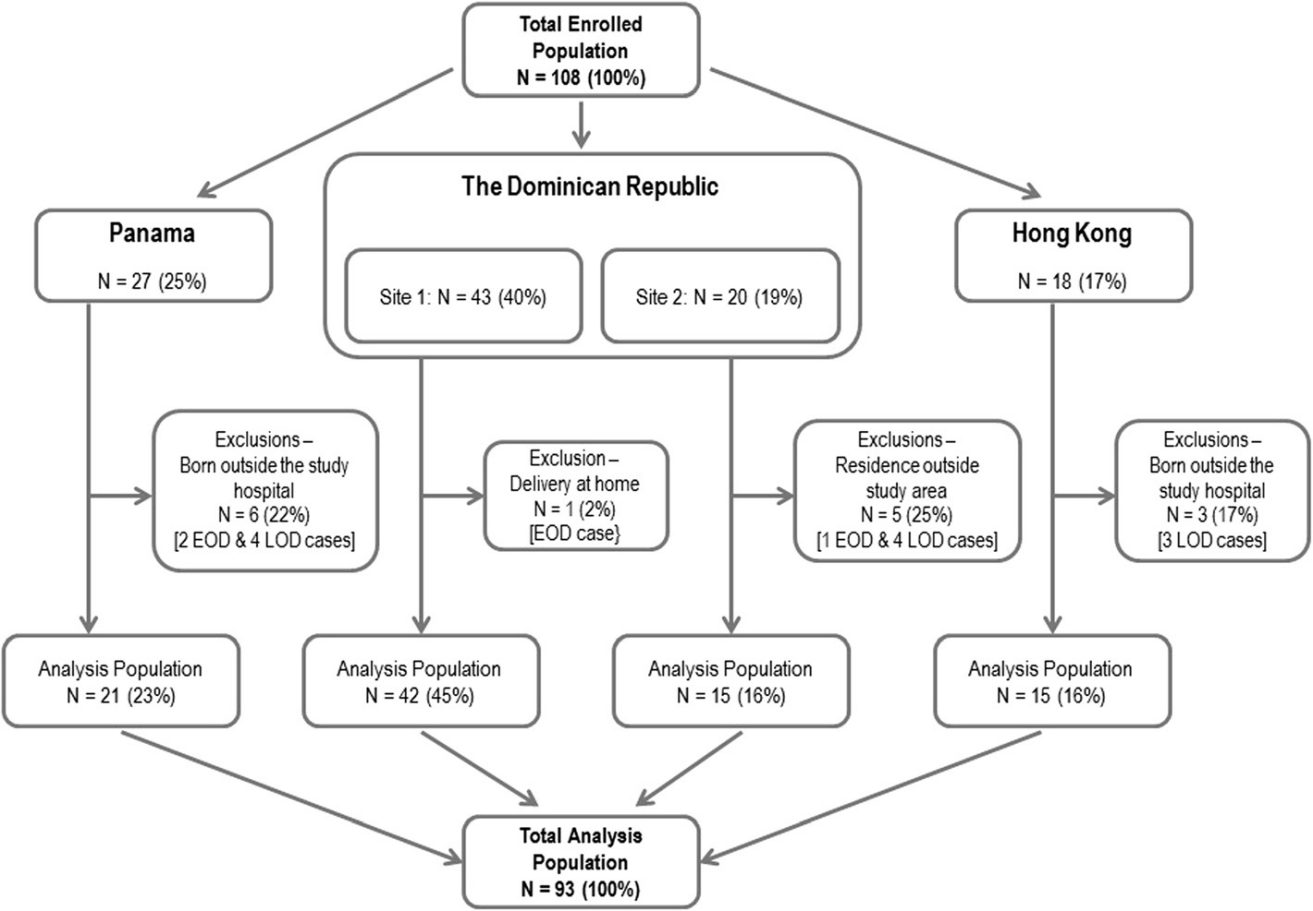
Participant distribution. Footnote: Four participants were retrospectively enrolled. EOD: Early Onset Disease; LOD: Late Onset Disease

Of the 108 participants enrolled with GBS culture confirmed disease, 93 met pre-defined eligibility criteria and were included in analyses (Analysis Population) (Fig. 1). Of the 15 exclusions, 10 were born outside the study hospital and five resided outside the study catchment area. Bangladesh did not identify any GBS cases over the study period.

Group B streptococcus was isolated from blood or CSF cultures in all but one case. A single case from Hong Kong had GBS isolated from both blood and joint fluid samples.

The Analysis Population (Table 1) represented diverse ethnic and racial origin groups reflecting the different geographic regions. The lowest mean birth weight was reported in Dominican Republic (2533 g) which may reflect the relatively high prematurity rate (40 %) and capture of high risk pregnancies through an obstetric referral center (Table 1).

**Table 1:** Demographic and other baseline characteristics of the study population per study site and overall

	Panama	Dominican Republic		Hong Kong	Total
		*Site 1*	*Site 2*		
*N*	21	42	15	15	93
Males/females	11/10	22/20	6/9	5/10	44/49
Racial origin/Ethnicity					
Asian/Chinese	-	-	-	15 (100 %)	15 (16 %)
Black	2 (10 %)	16 (38 %)	1 (7 %)	-	19 (20 %)
Caucasian	-	-	-	-	-
Hispanic	18 (86 %)	26 (62 %)	14 (93 %)	-	58 (62 %)
Other	1 (5 %)^*^	-	-	-	1 (1 %)
Birth weight (grams)					
Mean birth weight (SD)	2819 (719)	2533 (825)	2855 (654)	3234 (397)	2762 (753)
Median (Range)	2950 (740–3900)	2665 (907–3964)	2954 (1000–3636)	3250 (2220–3850)	2950 (740–3964)
Gestational age (weeks)					
Mean gestational ageδ (SD)	38 (3)	37 (4)	38 (3)	39 (1)	37 (4)
Median (Range)	38 (25–41)	38 (26–42)	39 (27–42)	39 (37–41)	38 (25–42)
Premature (<37 wks)	5 (24 %)	18 (43 %)	1 (7 %)	0(0 %)	24 (26 %)
Premature 34-37wks	4 (19 %)	10 (24 %)	0		14 (15 %)
Very premature <34 weeks	1(5 %)	8 (19 %)	1 (7 %)		10 (11 %)
Mother intrapartum antibiotic administered					
Yes	2 (10 %)	7 (17 %)	-	1 (7 %)	10 (11 %)
No	17 (81 %)	35 (83 %)	15 (100 %)	14 (93 %)	81 (87 %)
Unknown	2 (10 %)	-	-	-	2 (2 %)

Other = Indian (*n* = 1); δ gestational age is defined as gestational age at the time of delivery

All study hospitals offered risk based screening for GBS rather than systematic screening for colonization in late pregnancy. A subset of GBS cases were born to mothers recognized as at clinical risk of GBS and subsequently treated during delivery with IAP: 17 % and 10 % of GBS cases from Dominican Republic and Panama, respectively (Table 1). The most common indication for IAP was prolonged rupture of the membranes with intravenous ampicillin most commonly prescribed. Without additional information on the duration and dose of the intravenous antibiotics administered, it is difficult to comment further on the reasons underlying the failure of this intervention. Between 92–100 % of EOD cases occurred in the first 48 h of life across all centers (Table 2). The distribution of age at the time of LOD admission differed by center: Hong Kong reported the lowest median and narrowest range of age at LOD admission (14 days; range of 9–20 days) compared to centers in Latin America (median 26 and 32 days; range of 8–57 and 18–85 days, respectively for Dominican Republic and Panama).

**Table 2:** Age at admission of the study population per study site

	Panama	Dominican Republic		Hong Kong	Total
		*Site 1*	*Site 2*		
EOD (N)	12	42	3	10	67
Median age (days) at admission (range) % admitted in first 48 hrs	0 (0–3) 92 %	0 (0–2) 98 %	0 (0–3) 67 %	0 (0–0) 100 %	0 (0–3) 96 %
LOD (N)	9	N/A	12	5	26
Median age (days) at admission (range)	32 (18–85)	N/A	25.5 (8–57)	14 (9–20)	20.5 (9–85)

### Incidence rate and case fatality ratio

The incidence of GBS varied across countries, but EOD was consistently more frequent than LOD (Table 3). Excluding Bangladesh, where no cases were identified, the incidences of EOD per 1000 births ranged from 0.76 (95 % CI: 0.41–1.79) in Hong Kong to 2.35 (1.74–3.18) in Dominican Republic. The incidence of LOD ranged from 0.17 (0.10–0.30) in Dominican Republic to 0.58 (0.31–1.10) in Panama. The highest case fatality rate (CFR) for GBS cases was recorded in Dominican Republic and the lowest in Hong Kong (Table 3).

**Table 3:** Incidence rate of invasive group B streptococcal disease and case fatality ratio in the study population***

GBS incidence rate												
	Panama			Dominican Republic*						Hong Kong		
				*Site 1*			*Site 2*					
	*EOD*	*LOD*	*Total*	*EOD*			*LOD*			*EOD*	*LOD*	*Total*
Observed GBS cases	12	9	21	42			12**			10	5	15
Live births	15 500	15 500	15 500	17 867			70 297			13 244	13 244	13 244
Incidence rate per 1 000 live births	0•77 (0•44–1•35)	0•58 (0•31–1.10)	1•35 (0•89–2•07)	2•35 (1•74–3•18)			0•17 (0•10–0•30)			0•76 (0•41–1•39	0•38 (0•16–0•88)	1•13 (0•69–1•87)
Number of days hospitalized												
Mean (SD)	11 (5)			9 (6)			6 (5)			16 (4)		
Median (Range)	11 (0–16)			10 (0–39)			6 (0–13)			15 (8–24)		
Case Fatality Ratio												
	*EOD*	*LOD*	*Total*	*EOD*	*LOD*	*Total*	*EOD*	*LOD*	*Total*	*EOD*	*LOD*	*Total*
Deaths	2	1	3	9	-	9	1	5	6	1	0	1
Number of cases	12	9	21	42	-	42	3	12	15	10	5	15
Case fatality ratio	16•7 % (4•7–44•8)	11•1 % (2•0–43•5)	14•3 % (5•0–34•6)	21•4 % (11•7–35•9)	-	21•4 % (11•7–35•9)	33•3 % (6•1–79•2)	41•7 % (19•3–68•0)	40•0 % (19•8–64•3)	10•0 % (1•8–40•4)	0 % (0–43•4)	6•7 % (1•2–29•8)

*An overall early and late onset disease (EOD and LOD) incidence rate for Dominican Republic was not calculated because Site 1 (maternity) used a hospital-based denominator and Site 2 (pediatric) used a community-based denominator. **The three EOD cases recorded at Site 2 (pediatric) were not included in incidence calculations as cases were from different hospital birth cohorts without a clear denominator. *** Incidence for Bangladesh is not represented as no cases were identified among 4227 births. However the 95 % confidence interval around the incidence estimate is 0–0.9 per 1000 LB

Although clinical signs and symptoms were not routinely collected within the study, review of GBS cases for which both blood and CSF specimens are available for the same individual can inform on clinical presentation; a positive CSF culture indicating meningitis. Both Blood and CSF cultures were available from 46 infants (Table 4). The four fatalities among these 46 participants were all associated with meningitis (three LOD in Dominican Republic and one EOD in Hong Kong).

**Table 4:** Case fatality ratio (%) by disease presentation and center for a study population subset with both blood and cerebrospinal fluid cultures available (*n* = 46)

Case Fatality Ratio												
	Panama			Dominican Republic						Hong Kong		
				*Site 1*			*Site 2*					
	*Bacteremia*	*Meningitis*	*Total*	*Bacteremia*	*Meningitis*	*Total*	*Bacteremia*	*Meningitis*	*Total*	*Bacteremia*	*Meningitis*	*Total*
Deaths	0	0	0	0	-	0	0	3	3	0	1	1
Number of cases	14	3	17	7	-	7	2	7	9	7	6	13
Case fatality ratio	0 % (0–21•5)	0 % (0–56•2)	0 % (0–18•4)	0 % (0–35•4)	-	0 % (0–35•4)	0 % (0–65•8)	42•9 % (15.8–75.0)	33•3 % (12•1–64•6)	0 % (0–35•4)	16•7 % (3•0–56•4)	78 % (1•4–33•3)

### Serotype distribution

**Fig. 2:**
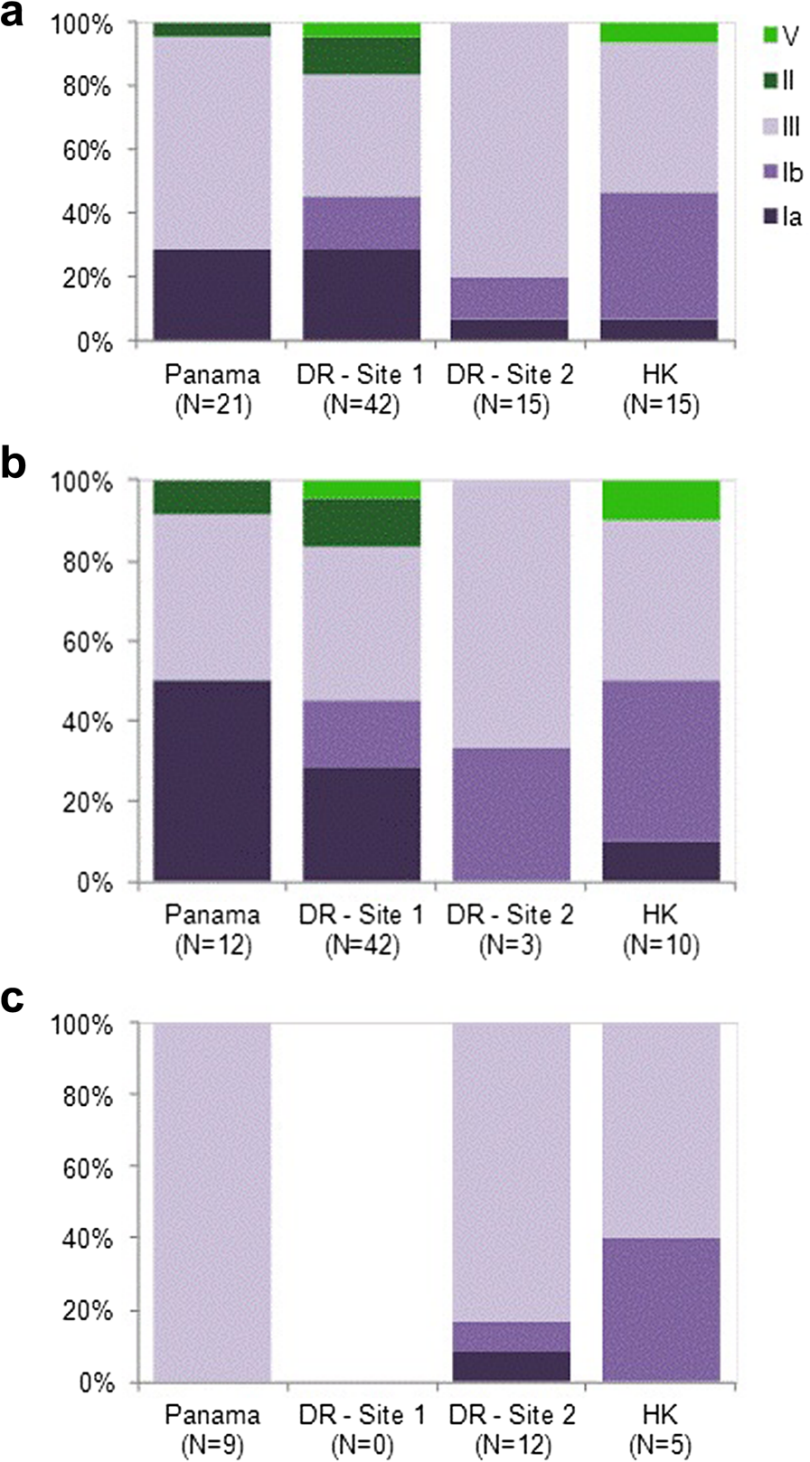
Serotype distribution by country and by center ((Analysis Population) for all GBS cases (**a**) for early onset disease (**b**) and late onset disease (**c**). Footnote: No late onset disease cases were recorded at Site 1 in the Dominican Republic as this was a maternity without a pediatric facility

Overall, 91 % of all GBS cases were due to serotypes Ia, Ib or III (87 % of all EOD; 100 % of all LOD; Fig. 2a). Serotype III alone was responsible for 40 % of EOD (Fig. 2b) and 85 % of LOD cases (Fig. 2c). Serotype distribution varied geographically. For EOD, serotype III predominated in Dominican Republic (38 %), while serotype Ia was most common in Panama (50 %) and in Hong Kong serotypes Ib and III were equally represented (40 % of disease each). For LOD, serotype III predominated in Panama and Dominican Republic (100 % and 83 % of cases respectively), while serotype Ib accounted for 40 % cases in Hong Kong. All fatal cases were serotype III. The serotype distribution differed between the two Dominican Republic hospital as one (maternity) captured only EOD cases and the second (pediatric) captured mainly LOD cases.

## Discussion

This study confirms the importance of GBS infection as a cause of early onset disease (EOD) in newborns. The incidence reported in the Dominican Republic (2.35 EOD per 1000 LB) was similar to the highest incidence reported in the literature from South Africa (2.0 per 1000 LB) [13, 14] and to rates reported from the United States before universal screening and IAP implementation (1.80 per 1000 LB) [15, 16]. EOD incidence was three-fold lower in Panama (0.76) and Hong Kong (0.77), while no cases were found in Bangladesh. The incidence in Hong Kong was higher than previous estimates from the Asian/Western Pacific region [2] highlighting the importance of having adequate clinical and microbiological diagnostics in place. The range of incidence estimates by country also prompts consideration of potential factors driving this variability.

The main risk factor for infant GBS disease is rectovaginal colonization of the mother during late pregnancy [1]. The high incidence of EOD in Dominican Republic may reflect the high reported prevalence (44 %) of maternal colonization in the population attending for delivery at study hospitals [17]*.* A lower maternal prevalence has been reported in Asia (7.7 %–10.4 % in Bangladesh and Hong Kong respectively [18, 19], though the limited variation between maternal data from Bangladesh and Hong Kong make it unlikely that colonization alone can drive the differences in observed disease incidence.

Racial origin, reflecting genetic or behavioral differences, may also contribute to the variation in disease incidence. Black racial origin is associated with higher GBS disease incidence [20–22]. Approximately 40 % of EOD cases in Dominican Republic were of black racial origin reflecting the potential contribution of that risk factor to disease incidence within that country setting. However, Dominican Republic also reported the highest proportion of preterm infants among GBS EOD cases (40 %), another reported risk factor for GBS disease [23]. It remains difficult to tease apart the individual contributions of these risk factors to overall disease incidence.

Case ascertainment is an important determinant of incidence and can be driven by access to healthcare, clinical practice and diagnostics [3]. Calculations for EOD incidence were based on hospital in-born birth cohorts, meaning all mothers had access to healthcare during the period of greatest risk for EOD. Indeed, over 90 % of EOD cases presented within the first 48 h of life across all study centers emphasizing the importance of establishing hospital based surveillance in place during this post-delivery period.

As an observational study, local clinical standards for the investigation of suspected sepsis were followed which may have introduced variation in case ascertainment. Indeed, a previous systematic review of neonatal sepsis etiology in developing countries reported a lower proportion of cases due to GBS in rural referral hospitals versus maternity hospitals (1.0 % versus 6.6 % of neonatal cases, respectively) [24]. All centers in the current study, other than Bangladesh, represented urban tertiary maternity centers. However, it was beyond the scope of the current study to directly assess the impact of clinical practice on reported incidence. The ideal may be to systematically take cultures on all infants admitted to hospital to further maximize case ascertainment, but this is often not practical within routine care. Unfortunately, data on the number of cultures taken during the study were not routinely collected to ascertain the impact of this aspect of clinical practice on case ascertainment.

The distribution of organisms causing neonatal sepsis at a center may also indicate differences in clinical and hygiene practices. Zhaidi et al. found a preponderance of *Staphylococcus aureus* and Gram negative organisms causing neonatal sepsis in studies from Asia [24]. These organisms are commonly thought to be environmentally-acquired, raising concerns that poor hygienic practices may mask vertically-acquired infections. The study center in Bangladesh mirrored this pattern with *Staphylococcus aureus* and *Klebsiella pneumoniae* being the most commonly isolated early infant pathogens (representing 2 % of all cultured blood samples). Future studies would benefit from a systematic description of all organisms found to responsible for neonatal sepsis to assess the potential impact of hygiene practices within and across sites.

The observational nature of this study may also have introduced variability through diagnostic and microbiology practice. For example, despite general recommendations across all study hospitals to collect 1–2 mL of blood for culture, blood volumes collected in infants may have varied which can influence GBS isolation rates [25]. In addition differences in antibiotic administration rates could have differentially affected GBS culture yields. Unfortunately, data on blood volumes and antibiotic administration were not routinely collected within the current study to assess the possible impact.

GBS isolation rates are reported to be higher with automated versus manual culture methods, and with selective versus non-selective culture methods [3, 26]. All centers used automated and enriched culture techniques which should have limited the impact of diagnostics on reported GBS incidence.

Reasons underlying the reported zero incidence of GBS disease in Bangladesh could include absence of disease in the population, although one GBS case was identified after study end, differences in hygiene practice or antibiotic usage leading to masking of GBS infection. As this study considered a hospital born birth cohort that remained in hospital for up to 3 days post-delivery, it is unlikely that poor healthcare seeking behavior can account for lack of EOD case identification within the study cohort. However, given the high proportion of births that occur at home in the Mizapur region (75 %), the study cohort may not be representative of the regional birth cohort. Access and cost of healthcare may have played a part in under-ascertainment of LOD cases. The relatively small birth cohort, compared to other study hospitals (up to 18,000 births) is reflected in the uncertainty of the incidence estimate (Table 3) and indicates a potential role of chance in the finding.

LOD rates were lower than EOD rates across all centers (0.17–0.58 vs 0.76–2.35 per 1000 LB). While this reflects the published literature [3] some under-estimation of LOD remains likely: some infants may have moved out of the hospital catchment area or presented to non-study hospitals, particularly in the urban study areas where a choice of pediatric services exists. Access to healthcare post-delivery will also impact LOD case ascertainment; most likely an issue in Bangladesh where the study hospital was the only hospital within a rural community. For infections that are fatal in the absence of prompt treatment, delays in reaching healthcare access would reduce case identification [10, 27].

Elsewhere, population movements may have led to an under-estimation of LOD. It is known that women from Haiti and the Chinese mainland may access delivery care in Dominican Republic and Hong Kong, respectively. Women returning to their homeland post-delivery will likely result in failure to identify LOD cases in the study cohort. Late onset disease incidence estimates based upon community population denominators (Dominican Republic) may also have under-estimated incidence. If there is differential use of pediatric services across Santo Domingo, perhaps for proximity reasons, it may not be appropriate to use the catchment population from the whole of city in incidence calculations. If one assumes that only infants living in the same sub-district of Santo Domingo as the study hospital presented with a sick infant, the estimated incidence of LOD per 1000 LB increases from 0.17 (95 % CI: 0.10–0.30) to 0.39 (95 % CI: 0.19–0.81) highlighting the complexity of assumptions underlying LOD incidence calculation.

This study showed the potential for high case fatality with GBS (7–40 % by center). The high CFR reported in Dominican Republic may have been driven by it being a tertiary center with high rates of preterm birth [23], as mortality was 29 % among preterm infants with GBS compared with 16 % among term infants with GBS. Meningitis may have been a key clinical factor associated with the high CFR reported among LOD cases in Dominican Republic, where 10 of 12 LOD cases presented with meningitis. This association of meningitis and fatality with invasive GBS disease, possibly as a result of infection with the highly virulent ST-17 type III clone, is in line with previous observations [13, 28, 29].

Overall 91 % of GBS disease cases were attributed to serotypes Ia, Ib and III, highlighting the potential impact of a vaccine targeting these serotypes. Future studies should also consider antibiotic resistance profiling of GBS isolates to obtain a clearer understanding of the relative impact of different interventions, including both screening and IAP, as well as a vaccine and immunization strategies.

## Conclusions

This study confirms the importance of GBS as a pathogen in young infants, but with considerable variation in incidence and CFR across countries. The study was not designed to assess the impact of differences in healthcare access, clinical practice, specimen processing, antibiotic usage or population differences. However, all centers reporting invasive GBS reported a preponderance of disease in the first two days of life emphasizing the importance of active surveillance within the newborn population. The reported incidence rates emphasize the need to perform larger observational studies with standardized methodologies for case ascertainment, laboratory analyses and clinical follow up to determine IAP and antibiotic use across a broad range of healthcare facilities, urban and rural, in different regions and countries.
